# Did Massachusetts COVID-19 vaccine lottery increase vaccine uptake?

**DOI:** 10.1371/journal.pone.0279283

**Published:** 2023-01-04

**Authors:** Yeunkyung Kim, Jihye Kim, Yue Li

**Affiliations:** 1 Department of Healthcare Administration and Policy, University of Nevada, Las Vegas, Nevada, United States of America; 2 Department of Epidemiology and Biostatistics, University of Nevada, Las Vegas, Nevada, United States of America; 3 Department of Public Health Sciences, Division of Health Policy and Outcomes Research, University of Rochester Medical Center, Rochester, New York, United States of America; Medical College of Wisconsin, UNITED STATES

## Abstract

**Objective:**

We evaluated whether the Massachusetts COVID-19 vaccine lottery increased vaccine uptake.

**Methods:**

We analyzed data from the Centers for Disease Control and Prevention COVID-19 Vaccine Tracker to identify total number of adults aged 18 to 64 who received at least first dose of the COVID-19 vaccine or who were fully vaccinated in Massachusetts, Connecticut, Rhode Island, New Jersey, and Vermont during the study period of March 6 –July 31, 2021. Each of the five states contributed 148 days of a daily report on cumulative number of vaccinated people, comprising 740 state-days as the total sample size. We conducted multivariable, state-day level difference-in-differences (DID) regression using a negative binomial regression model that compared the change in outcomes for Massachusetts to those of four geographically adjacent comparison states without the lotteries, before and after the Massachusetts vaccine lottery announcement (June 15, 2021). Our analyses controlled for key state-level characteristics obtained from the American Community Survey as well as day fixed-effects to capture secular trends in the outcomes.

**Results:**

Massachusetts COVID-19 vaccine lottery was not associated with a significant increase in the number of adults aged 18 to 64 who were fully vaccinated or received at least one dose of the vaccine, compared with other states [Full dose, incidence rate ratio (IRR): 1.04, 95% confidence interval (CI): 0.97 to 1.11, P > 0.05; At least one dose, IRR: 0.99, 95% CI: 0.93 to 1.06, P > 0.05].

**Conclusions:**

There was insufficient evidence to conclude that Massachusetts COVID-19 vaccine lottery was associated with increased number of adult COVID-19 vaccinations.

## Introduction

Vaccination is one of the most important tools to help end the COVID-19 pandemic. COVID-19 vaccines are highly effective at preventing serious infections, hospitalizations, and deaths from the virus [1–3]. However, after widespread COVID-19 vaccine uptake from late 2020 to the middle of April 2021, the vaccination rate has slowed down across the US [4].

Many states since then have implemented COVID-19 vaccine incentive programs to increase vaccination rates. On June 15 of 2021, the state of Massachusetts announced COVID-19 vaccine lottery to boost vaccine uptake in the state. While about 60% of residents have been vaccinated in Massachusetts by May of 2021, the pace of vaccinations has slowed since then [5]. The vaccine lottery had five weekly statewide drawings occurred running from July 26 to August 23, 2021 (Table 1). Massachusetts residents 18 years or older who received two doses of Pfizer/Moderna or one dose of Johnson and Johnson were eligible to enter the lottery to win one of five $1 million cash prizes. In addition, fully vaccinated residents between the ages of 12 and17 may enter for the chance to win one of five, $300,000 scholarship grants.

**Table 1:** Massachusetts COVID-19 lottery registration deadline, drawing date, and announcement date.

Registration Deadline	Drawing Date	Announcement Date
July 22	July 26	July 29
July 29	August 2	August 5
August 5	August 9	August 12
August 12	August 16	August 19
August 19	August 23	August 26

Note: Full vaccinations (e.g., two doses of Pfizer/Moderna or one dose of Johnson and Johnson) required to enter the lottery by each deadline above. All dates are in 2021.

Previous research on the efficacy of financial incentives for increasing vaccine uptake such as COVID-19 vaccine lotteries is limited. For instance, a recent study reported that a lottery-based incentive in Ohio was not associated with increased COVID-19 vaccination rates among adults [6]. Experts have suspected that the lack of effectiveness of such programs may be due to the fact that a lottery-based incentive does not directly address vaccine hesitancy and access barriers to vaccinations [7]. Nevertheless, more evidence on the effectiveness of different state vaccine lotteries, or lack thereof, is needed to make definite conclusions on the efficacy of state COVID-19 vaccine lottery programs. To date, research on the effect of Massachusetts COVID-19 vaccine lottery on adult vaccination rates has not been explored.

We sought to examine whether Massachusetts’s large cash lottery drawings that benefit only a few lucky winners were able to motivate more people for vaccine uptake. In this study, we compared the change in the number of adult COVID-19 vaccinations before and after the Massachusetts vaccine lottery announcement with those of states that did not launch any COVID-19 vaccine lotteries that award large financial prizes.

## Methods

This study was approved by the institutional review board of the University of Rochester, and informed consent was waived because this study was conducted using state-level datasets without any patient information involved.

### Study sample

We analyzed data from the Centers for Disease Control and Prevention (CDC) COVID-19 Vaccine Tracker [8] to identify total number of people who received at least first dose of the COVID-19 vaccine or who were fully vaccinated (e.g., have second dose of a two-dose vaccine or one dose of a single-dose vaccine) based on where recipient lives. Data represent overall US COVID-19 vaccine deliveries and administration at national and jurisdiction levels at all vaccine partners (e.g., jurisdictional partner clinics, retail pharmacies, and long-term care facilities).

Our study sample consisted of adults aged 18 to 64 who received at least one dose of the vaccine or who were fully vaccinated in Massachusetts, or in four comparison states (Connecticut, Rhode Island, New Jersey, and Vermont) during the study period of March 6 –July 31, 2021. The four comparison states were chosen because of their geographic proximity to Massachusetts and because they did not launch the COVID-19 vaccine lotteries that awarded large cash prizes during the study period [9]. These comparison states were previously used to evaluate a major health reform in Massachusetts in the literature [10]. We limited the study period to July 31 because government moved forward requiring employees to be vaccinated late July or early August [11, 12], potentially affecting adult vaccinations in Massachusetts and the comparison states. This study period provided sufficient pre- and post-Massachusetts vaccine lottery announcement (June 15, 2021) periods for each state to keep track of total number of residents who are vaccinated. Each of the five states contributed 148 days (March 6 to July 31) of a daily report on cumulative number of people who received vaccine, thus comprising 740 state-days as the total sample size.

### Outcome measures

In this study, our primary outcome measure was the daily cumulative number of fully vaccinated adults aged 18 to 64 in either Massachusetts or each of other four comparison states, where full vaccination was defined as receiving two doses of Pfizer/Moderna or one dose of Johnson and Johnson. Our secondary outcome measure was the daily cumulative number of those who received at least one dose of the COVID-19 vaccine in either Massachusetts or other comparison states.

### Statistical analysis

We conducted multivariable, state-day level difference-in-differences (DID) regression that compared the change in outcomes for Massachusetts to those of four comparison states before and after the Massachusetts vaccine lottery announcement. The effect of Massachusetts lottery program on the daily cumulative number of vaccinations was estimated using a negative binomial regression model with exposure of the number of state population and a log link function. Since the outcomes were the daily cumulative number of vaccinated adults, the mean of outcomes increases over time and the variance of outcomes increases faster over time. Thus, the outcomes in this study were overdispersed. Therefore, we chose the negative binomial regression model over the Poisson regression model to allow the conditional variance of the outcome to be greater than its conditional mean, providing greater flexibility in model fitting [13]. The model structure is as follows:

$${Log\;(Y}_{sd})=\beta_{0}+\beta_{1}*{MA}_{s}+\beta_{2}*{Post}_{d}+\beta_{3}*({MA}_{s}*{Post}_{d})+X_{s}^{\prime }\beta_{4}+\beta_{5}*{Day}_{d}+\varepsilon_{sd}.$$ (1)

The dependent variable in Eq (1) (*Y*_*sd*_) was cumulative number of vaccinated adults in state *s* in date *d*. The variable (*MA*_*s*_) was a binary indicator which was equal to the value 1 for Massachusetts and the value 0 for four comparison states. The variable (*Post*_*d*_) was a binary indicator which was equal to the value 1 for the post-announcement period (June 15—July 31, 2021) and the value 0 for the pre-announcement period (March 6 –June 14, 2021). The coefficient *β*_1_ estimated the expected difference in log count of vaccinated adults between Massachusetts and other four comparison states before the announcement, whereas *β*_2_ estimated the expected difference in log count of vaccinated persons in the comparison states before and after post-announcement. The main parameter of interest was the coefficient on the interaction term (*β*_3_), which estimated the expected change in log count of vaccinated adults in Massachusetts, compared to those in four comparison states, before and after lottery announcement.

A positive and significant coefficient on the interaction term would indicate that Massachusetts COVID-19 vaccine lottery was significantly associated with increased number of people vaccinated compared with other four comparison states. All results are reported as incidence rate ratios (IRRs). Data were analyzed using Stata version 16 (StataCorp) [14]. A two-sided *p* < 0.05 was considered statistically significant.

### Covariates

The regression presented in Eq (1) included a set of variables that could have affected changes in the number of vaccinated persons (represented by $X_{s}^{\prime }$). Our analyses controlled for key state-level characteristics including percentage of non-Hispanic white population, percentage of those speaking language other than English, and median household income. These variables were obtained from the 2020 American Community Survey, conducted by the U.S. Census Bureau [15]. We further controlled for day fixed-effects to capture secular trends in the outcomes.

## Results

**Fig 1:**
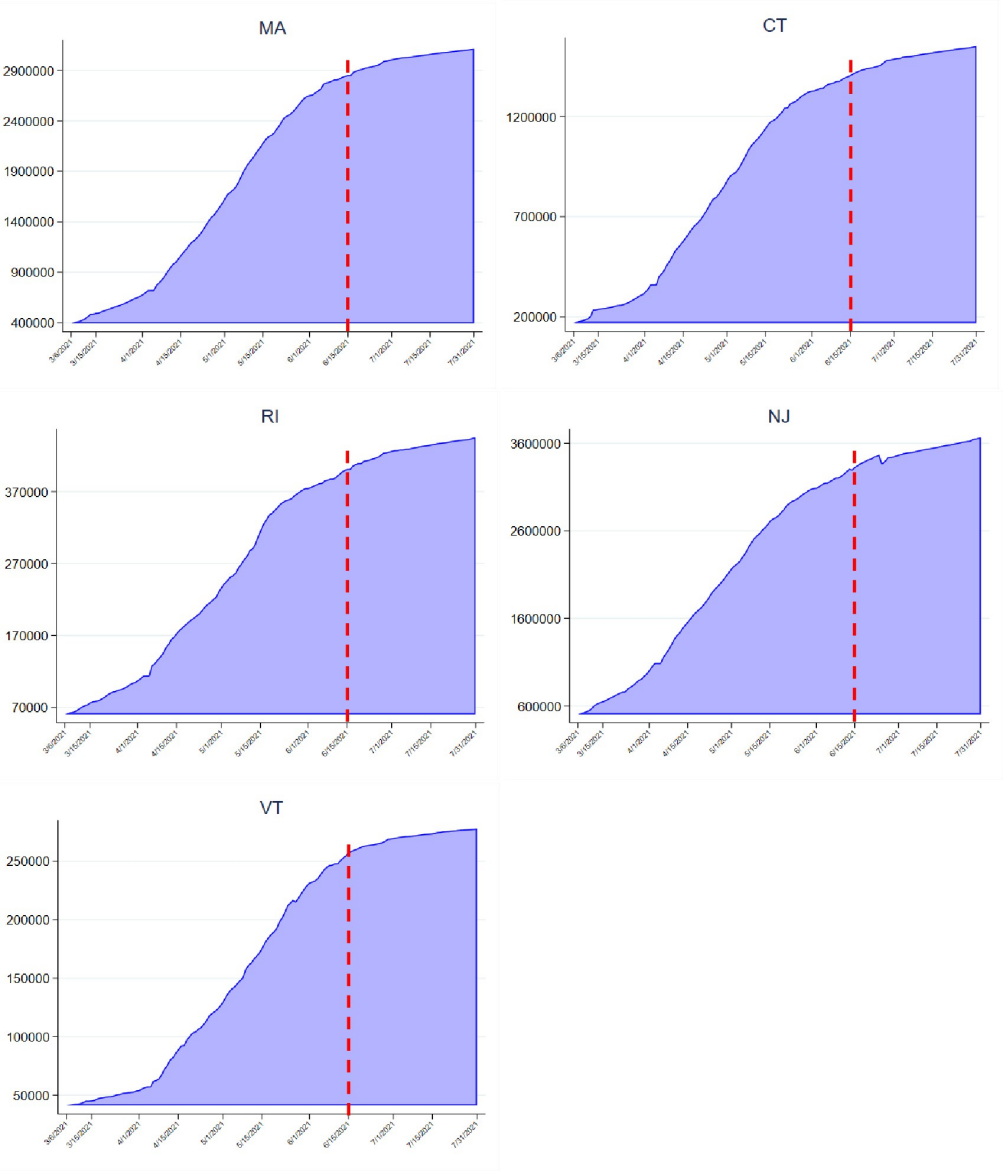
Cumulative number of fully vaccinated adults aged 18 to 64 in Massachusetts and other four comparison states. Abbreviations: MA, Massachusetts; CT, Connecticut; RI, Rhode Island; NJ, New Jersey; VT, Vermont. Note: Vertical dash line indicates Massachusetts COVID-19 Vaccine announcement date. Source: Author’s analysis of the Centers for Disease Control and Prevention COVID-19 Vaccine Tracker.

Fig 1 presents the daily cumulative number of fully vaccinated adults in Massachusetts and other four comparison states. Massachusetts experienced a sharp increase in the number of people fully vaccinated until late May of 2021 but the rate of vaccinations has slowed down since early June despite the vaccine lottery announcement. Although the numbers of fully vaccinated adults differed by states, similar trends were observed in Massachusetts and other comparison states.

Table 2 presents descriptive statistics for state-level characteristics in 2020. Among the states, New Jersey had the largest population, followed by Massachusetts. Percentage of non-Hispanic white population was highest in Vermont but lowest in New Jersey. Percentage of those speaking language other than English was lowest in Vermont. Lastly, median household income was higher in Massachusetts and New Jersey than other states.

**Table 2:** Descriptive statistics for state-level characteristics in 2020.

	MA	CT	RI	NJ	VT
Population, N	6,893,574	3,557,006	1,057,125	8,882,371	623,347
Non-Hispanic white, %	70.3	65.6	70.8	54.3	92.5
Speaking language other than English, %	24.7	22.5	22.7	32.2	5.5
Median household income, $	85,843	78,833	71,169	85,751	63,001
Days before MA lottery announcement, N	101	101	101	101	101
Days after MA lottery announcement, N	47	47	47	47	47

Abbreviations: MA, Massachusetts; CT, Connecticut; RI, Rhode Island; NJ, New Jersey; VT, Vermont.

Note: N is number. % is column percentage.

Source: Author’s analysis of 2020 American Community Survey.

Table 3 shows the results of multivariable state-day level DID estimates of Massachusetts COVID-19 vaccine lottery on the number of vaccinations. After controlling for state-level covariates and day effects, Massachusetts COVID-19 vaccine lottery was not associated with a significant increase in the number of adults aged 18 to 64 who were fully vaccinated or received at least one dose of the vaccine, compared with other adjacent states [Full dose, IRR: 1.04, 95% confidence interval (CI): 0.97 to 1.11, P > 0.05; At least one dose, IRR: 0.99, 95% CI: 0.93 to 1.06, P > 0.05]. The regression confirmed that changes in the number of people who received vaccinations in Massachusetts after the lottery announcement were closely similar to those of comparison states.

**Table 3:** Results of state-day level difference-in-differences regression estimates of Massachusetts COVID-19 vaccine lottery on the number of vaccinations among adults 18 years or older.

	Full dose (n = 740)		At least one dose (n = 740)	
	IRR	95% CI	IRR	95% CI
MA x Post	1.04	0.97, 1.11	0.99	0.93, 1.06
MA	1.01	0.83, 1.23	1.04	0.85, 1.26
Post	0.56^***^	0.54, 0.59	0.59^***^	0.56, 0.62
State-level characteristics				
Speaking language other than English	1.00	0.98, 1.02	0.99	0.97, 1.01
Non-Hispanic white	1.00	0.98, 1.02	1.00	0.98, 1.02
Median household income	1.00	1.00, 1.00	1.00	1.00, 1.00
Dayⴕ	1.02^***^	1.02, 1.02	1.01^***^	1.01, 1.01

Abbreviations: IRR, incidence rate ratio; CI, confidence interval; MA, Massachusetts.

Source: Author’s analysis of the Centers for Disease Control and Prevention COVID-19 Vaccine Tracker and 2020 American Community Survey.

ⴕDay indicates days of a daily report on cumulative number of people who received vaccinations. Each of the five states contributed 148 days (March 6 to July 31, 2020), thus compromising 740 state-days as the total sample size.

*** p < 0.001

## Discussion

There was insufficient evidence in our analyses that the number of adult COVID-19 vaccinations between Massachusetts and other four comparison states differed before and after Massachusetts COVID-19 vaccine lottery. The COVID-19 vaccine lotteries that awarded large but uncertain financial prizes for a few selected winners may not be an effective means to encourage people to be vaccinated.

Our findings are consistent with the current literature on the association of states COVID-19 vaccine lotteries with vaccine uptake. Similar to our findings, a prior study [6] has found that after the state of Ohio announced a lottery system to pay randomly selected vaccine recipients up to $1 million, this lottery-based incentive was not associated with increased adult COVID-19 vaccination rates in Ohio. In contrast, that study found that after the lottery announcement, the slower decline in vaccinations in the US than in Ohio could be explained by expanded vaccine eligibility to adolescents, which was associated with an increase in adult vaccinations [6]. Similar to our findings, another recent article [16] showed that using data from the Johns Hopkins University Vaccine Tracker between April 28 and July 1, 2021, the large cash lotteries tied to COVID-19 vaccination in 19 states were not associated with increased number of vaccinations, when compared to states that did not have COVID-19 vaccine lotteries. Massachusetts was one of the 19 states but comparing the effects of Massachusetts COVID-19 vaccine lottery to the rest of the US without lotteries may not be an ideal comparison given different state-level characteristics and geographic proximity. Also, given that Massachusetts announced the lottery on June 15, 2021, two weeks before the end of the observation period in the study using the Johns Hopkins University Vaccine Tracker, it was likely to be too short to observe meaningful changes in vaccination rates in Massachusetts. This study contributes to this limited literature on the lottery-based incentive effects on the COVID-19 vaccine uptake in Massachusetts and provides further evidence that significant association was not found between the lottery-based incentive and the number of vaccinations.

Our findings demonstrate that lottery-style drawings may not be a promising strategy to address access barriers to vaccinations. Several approaches might be considered to boost vaccine uptake. One option is to use guaranteed small financial incentives that help offset expenses related to utilities such as electricity and water. For instance, a recent article [7] reported that in a pilot program in North Carolina, guaranteed a $25 cash card to adults who either received or drove someone to receive their first dose of COVID-19 was associated with slower decline in vaccination rate. A guaranteed small financial incentive may be far more effective, and less costly, in encouraging people to be vaccinated than drawings. Also, it is important to build up positive attitude toward COVID-19 vaccine to increase vaccine uptake. In particular, vaccine misinformation is rapidly spread by social media leading to vaccine hesitancy [17]. Therefore, health misinformation associated with COVID-19 vaccine should be addressed across social media platforms to boost vaccine uptake.

We acknowledge the limitations of our study. First, there may be potential uncontrolled characteristics that confound the estimated effect of Massachusetts COVID-19 vaccine lottery. For example, we do not have data on individual-level characteristics that may be associated with vaccine hesitancy. Second, the study findings are subject to the accuracy of data from the CDC COVID-19 Vaccine Tracker [8]. Finally, our analyses were based on daily report of number of vaccinations across five states during the study period, thus we end up having small sample size (n = 740), leading to potential loss of power to observe statistically significant association [18, 19].

## Conclusions

In conclusion, this study found that vaccination rates were not significantly different for Massachusetts compared to other adjacent states before and after Massachusetts COVID-19 vaccine lottery announcement, suggesting the lack of effect of the lottery on boosting vaccine uptake rate. Alternative approaches with guaranteed financial incentives or that directly address vaccine hesitancy should be considered in future policy interventions.

## Supporting information

S1 Data (XLS)
